# Differences in Outflow Facility Between Angiographically Identified High- Versus Low-Flow Regions of the Conventional Outflow Pathways in Porcine Eyes

**DOI:** 10.1167/iovs.64.3.29

**Published:** 2023-03-20

**Authors:** Clemens A. Strohmaier, Fiona S. McDonnell, Xiaowei Zhang, Daniel Wanderer, W. Daniel Stamer, Robert N. Weinreb, Alex S. Huang

**Affiliations:** 1 Department of Ophthalmology and Optometry, Kepler University Hospital, Johannes Kepler University, Linz, Austria; 2 Hamilton Glaucoma Center, The Viterbi Family Department of Ophthalmology, Shiley Eye Institute, University of California, San Diego, California, United States; 3 Department of Ophthalmology, Duke University, Durham, North Carolina, United States; 4 Department of Biomedical Engineering, Duke University, Durham, North Carolina, United States

**Keywords:** aqueous angiography, trabecular meshwork, aqueous humor outflow

## Abstract

**Purpose:**

To investigate differences in outflow facility between angiographically determined high- and low-flow segments of the conventional outflow pathway in porcine eyes.

**Methods:**

Porcine anterior segments (*n* = 14) were mounted in a perfusion chamber and perfused using Dulbecco's phosphate buffered solution with glucose. Fluorescein angiography was performed to determine high- and low-flow regions of the conventional outflow pathways. The trabecular meshwork (TM) was occluded using cyanoacrylate glue, except for residual 5-mm TM areas that were either high or low flow at baseline, designating these eyes as “residual high-flow” or “residual low-flow” eyes. Subsequently, outflow was quantitatively reassessed and compared between residual high-flow and residual low-flow eyes followed by indocyanine green angiography.

**Results:**

Fluorescein aqueous angiography demonstrated high-flow and low-flow regions. Baseline outflow facilities were 0.320 ± 0.08 and 0.328 ± 0.10 µL/min/mmHg (*P* = 0.676) in residual high-flow and residual low-flow eyes before TM occlusion, respectively. After partial trabecular meshwork occlusion, outflow facility decreased to 0.209 ± 0.07 µL/min/mmHg (−32.66% ± 19.53%) and 0.114 ± 0.08 µL/min/mmHg (−66.57% ± 23.08%) in residual high- and low-flow eyes (*P* = 0.035), respectively. There was a significant difference in the resulting IOP increase (*P* = 0.034).

**Conclusions:**

Angiographically determined high- and low-flow regions in the conventional outflow pathways differ in their segmental outflow facility; thus, there is an uneven distribution of local outflow facility across different parts of the TM.

Intraocular pressure (IOP) is the most important risk factor for glaucoma, and IOP lowering is still the mainstay for glaucoma treatment.^1^ The Goldmann equation expresses steady-state IOP as the balance between aqueous humor production and outflow facility with episcleral venous pressure as an additive factor.^2^ Thus, for the determination of steady-state IOP in this model, trabecular outflow facility is reduced to a single number. This simplification, however, neglects the spatial variability of aqueous humor outflow (AHO).

Several clinical and experimental studies have demonstrated the segmental nature of AHO. In many species (e.g., studies in postmortem eyes of pig, cow, cat, dog, and human; studies in living organisms such as cat, dog, non-human primates, and humans), segmental AHO has been shown via spatial differences in AHO tracer uptake in the trabecular meshwork (TM) using perfused microbeads,^3^^–^^5^ TM electron microscopic analyses after cationic ferritin perfusion,^6^^,^^7^ and after imaging of intracameral soluble tracers observed on the ocular surface using aqueous angiography.^8^^–^^15^ Regions with higher versus lower AHO have been shown to differ in their extracellular matrix composition, expressed proteins, and level of TGFβ-related profibrotic pathway activity.^9^^,^^16^

Segmental outflow may have direct clinical relevance because of the mechanism of action and inconsistent clinical response of many minimally invasive glaucoma surgeries (MIGSs). Trabecular outflow resistance accounts for 50% to 75% of totutlookflow resistance in the eye,^17^^–^^19^ and TM outflow resistance is increased in glaucoma.^20^^,^^21^ Several MIGS procedures target the TM; however, IOP reduction is variable and modest with these procedures.^22^^,^^23^ Mechanical TM bypass can change total TM outflow facility, but such devices are placed only in specific locations. It is not known which locations should be preferentially treated.^24^^,^^25^ In other words, current surgical procedures do not take into account the segmental nature of conventional outflow for placement of an incision or a device.

Thus, a conceptual gap arises between the Goldmann equation (where outflow facility is assumed to be uniform across the TM circumference) and imaging data, because facility is reduced to one number in the Goldmann equation whereas imaging data show segmental TM AHO with presumed differences in local outflow facility. However, an alternative explanation of segmental patterns in tracer studies is a segmental interaction between the TM and the tracer that is unrelated to outflow facility.

In the present study, we set out to estimate segmental TM outflow facility. We use porcine eyes as an initial investigation to this question to be complementary to prior work^26^ and because porcine eyes are considered a valid model for humans given similar physiology and outflow facility. To do so, residual AHO facility was assessed after isolating TM associated with angiographically determined baseline high- and low-flow regions in order to evaluate if different regions of the TM have different outflow facilities.

## Methods

Porcine eyes (*n* = 14, sourced from Yorkshire Crossbreed pigs, weight 80–110 kg, equal sex distribution) were obtained from an abattoir (shipped on blue ice within 24 hours of death; Sierra for Medical Science, Inc., Whittier, CA, USA) and trimmed of excess tissue upon arrival. After a 2-minute submersion in betadine, the eyes were stored in balanced salt solution (BSS) at 4°C.

### Tissue Preparation and Anterior Segment Perfusion Setup

**Figure 1.**
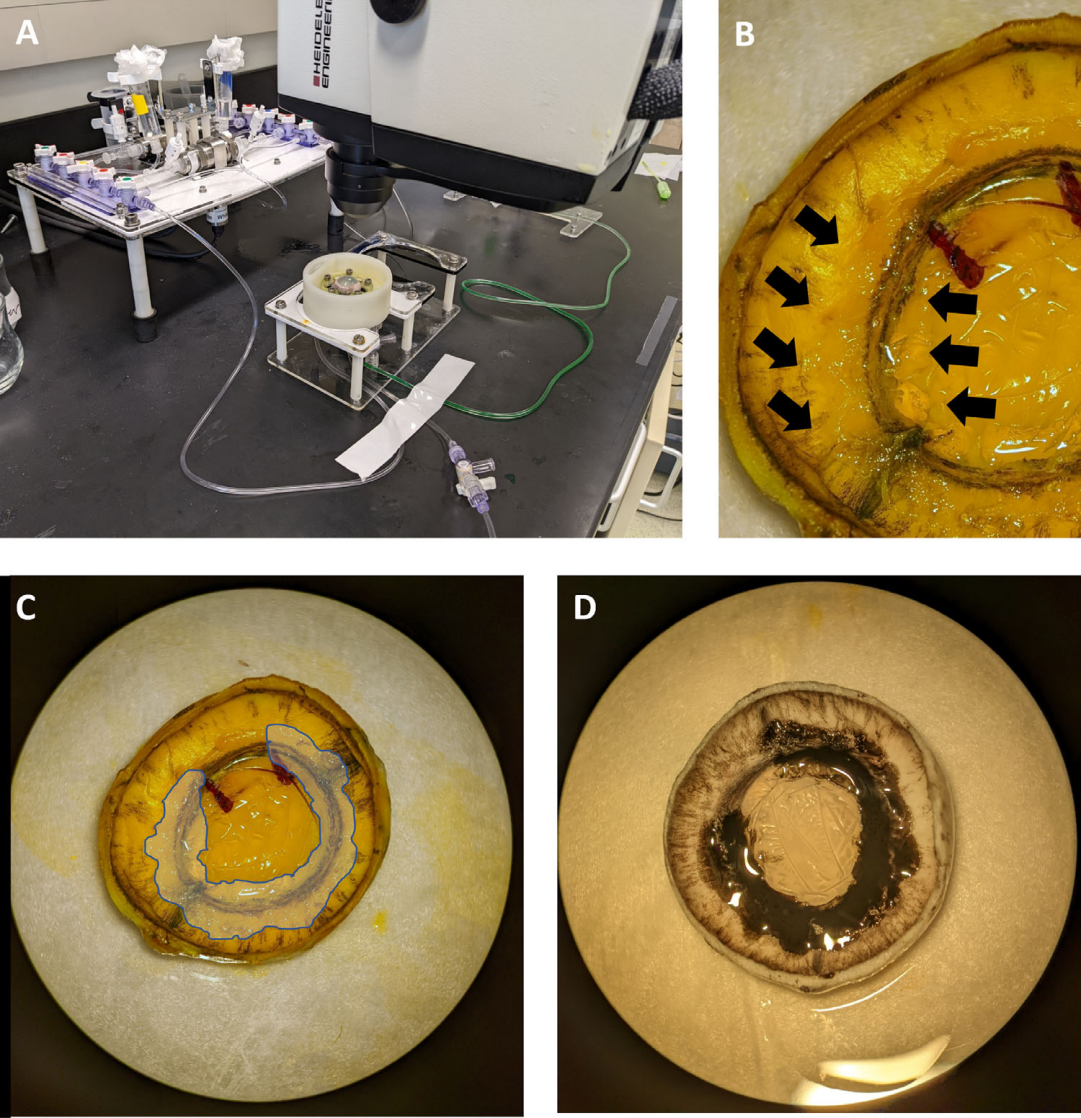
**(A)** Experimental setup. An anterior segment perfusion chamber was imaged using a Heidelberg SPECTRALIS Flex multimodal imaging device suitable for fluorescein and indocyanine green (ICG) angiography. (**B**–**D**) Inverted porcine anterior segment visualized under the microscope. (**B**) and (**C**) show the same eyes at different magnifications; the *arrows* and *shaded area* highlight the edges of the glued areas of the outflow pathways (change in texture). (**D**) For the purpose of illustration, colored cyanoacrylate glue was used in this eye to highlight the occluded TM.

**Figure 2.**
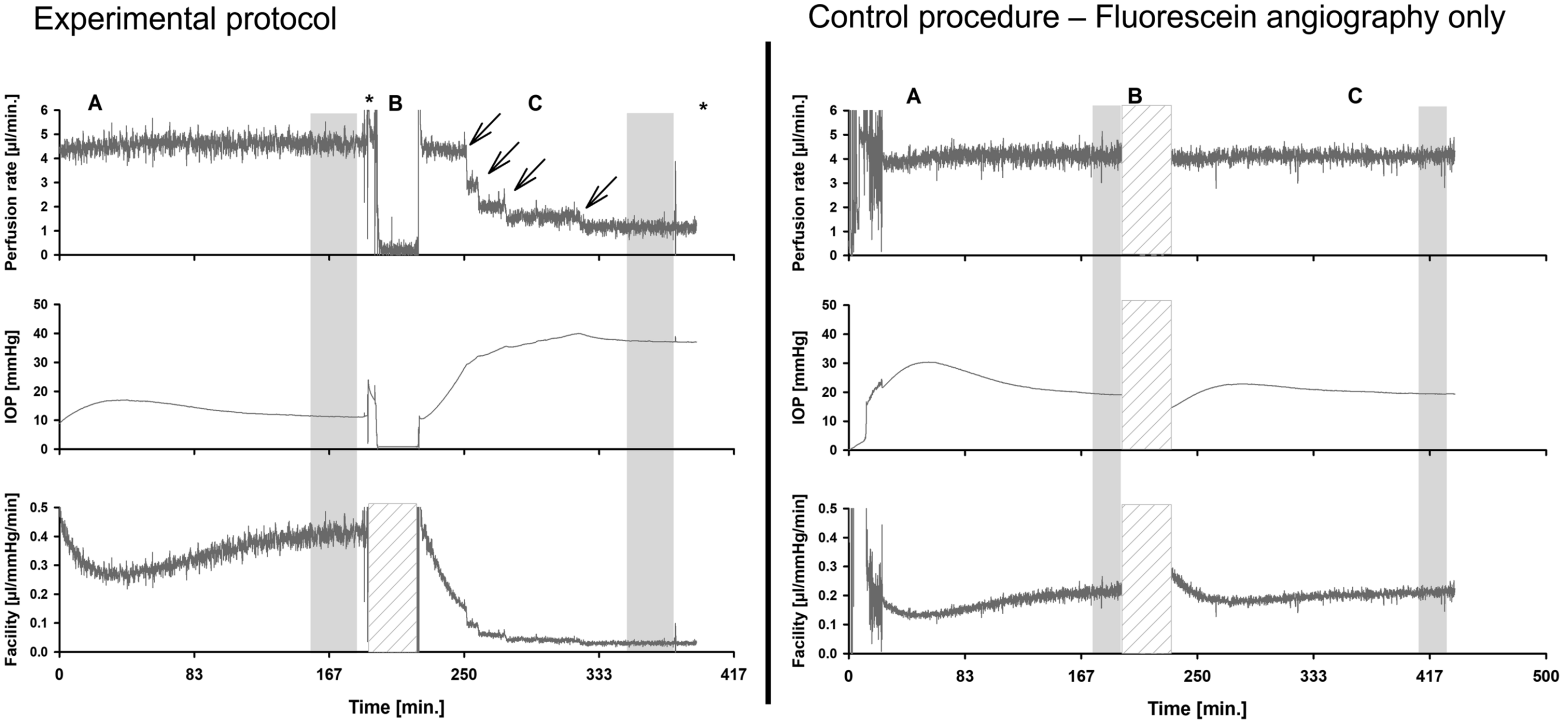
(*Left panel*) Representative tracing of the experimental protocol with the perfusion rate, IOP, and outflow facility recorded. (**A**) Perfusion at 4.5 µL/min with initial baseline. At the end of the baseline period, fluorescein angiography (*asterisk*) was performed to identify high- and low-flow segments in the AHO pathways. (**B**) Unclamping and TM occlusion using cyanoacrylate glue (facility tracing has been deleted for this section due to signal noise). (**C**) Re-clamping and stabilization. In some eyes, the perfusion rate had to be adjusted to keep IOP within the allowable pressure range of the device setup (*arrows*). At the end of this period, ICG angiography was performed (*asterisk*). Data in the *shaded gray areas* were analyzed. (*Right panel*) Control condition, where fluorescein angiography was performed with unclamping and clamping but no glue was applied.

The eyes were bisected at the equator to isolate the anterior segment. From the anterior segment, the vitreous, anterior retina, lens, and iris were carefully dissected to expose the trabecular meshwork. The anterior segment preparations were rinsed with BSS, and any remaining pigment was gently removed with sterile cotton tips. The anterior segments were then mounted onto an anterior segment perfusion chamber (iOnly Human, iPerfusion; Imperial College London, London, UK) at room temperature (Fig. 1).^27^ The perfusion system includes an anterior segment perfusion chamber connected to a syringe pump (PHD Ultra; Harvard Apparatus, Holliston, MA, USA) with an interposed flowmeter (SLI-0430; Sensirion AG, Stäfa, Switzerland). The chamber also contained a reference well with liquid at the same height as the limbus of the eye with a pressure transducer (Omegadyne PX409; Omega Engineering, Norwalk, CT, USA) placed between the reference well and eye. After the system was filled with sterile-filtered Dulbecco's PBS (DPBS) with glucose (1 g/L; Thermo Fisher Scientific, Waltham, MA, USA), the syringe pump was engaged to perfuse the anterior segment at 4.5 µL/min.^28^ The eyes were perfused until a stable baseline was achieved (Fig. 2), and only eyes with a baseline facility (calculated as perfusion rate divided by IOP) between 0.2 and 1.0 µL/mmHg/min were used for the study.^29^

### Experimental Protocol

**Figure 3.**
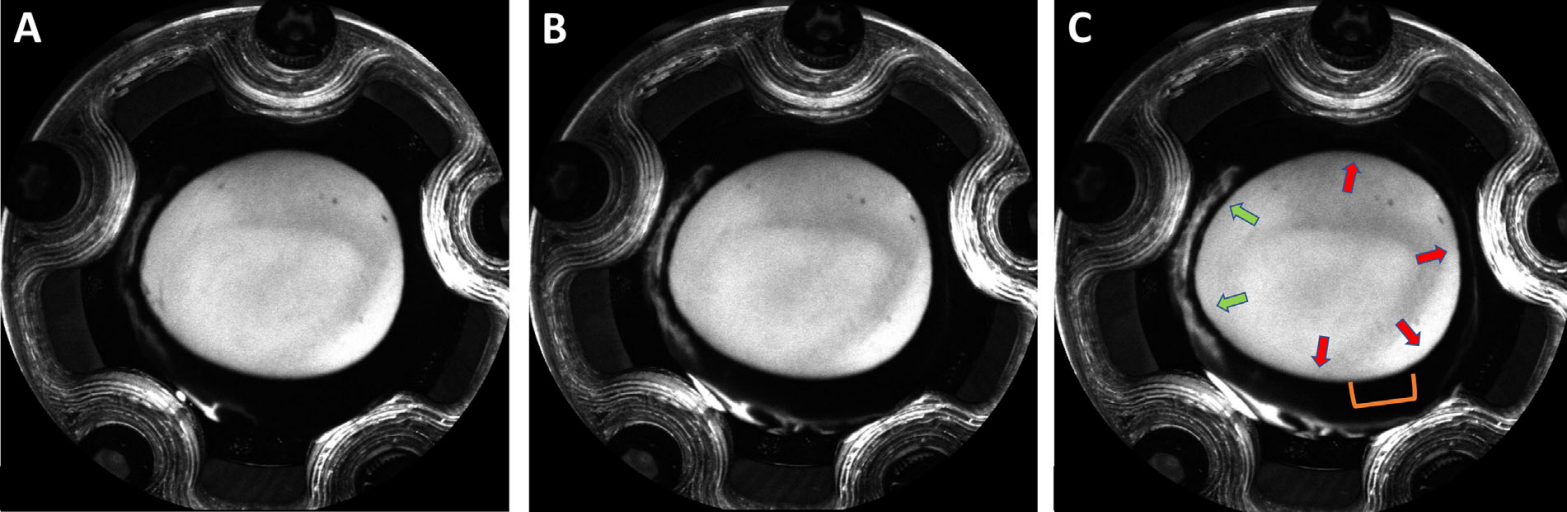
Determination of high- and low-flow regions using fluorescein angiography. (**A**–**C**) The time course of the fluorescein signal, with images taken at 30 seconds (**A**), 60 seconds (**B**), and 90 seconds (**C**). The image at 90 seconds (**C**) was used for the discrimination of high- and low-flow segments. High-flow (*green arrows*) and low-flow (*red arrows*) regions are indicated in **C**. The *orange bracket* marks the selected residual low-flow region being investigated in this eye (i.e., the remaining circumference except for this segment was occluded using cyanoacrylate glue).

After a stable baseline was achieved, the perfusion setup was flushed with fluorescein (2.5%; Akorn Pharmaceuticals, Gurnee, IL, USA) with the anterior chamber open to atmospheric pressure. The eye was pressurized again, and AHO images were obtained as described previously.^9^^,^^10^^,^^30^ In brief, a confocal laser scanning ophthalmoscope (SPECTRALIS Flex, Heidelberg Engineering, Heidelberg, Germany) with built-in filters for fluorescein and indocyanine green (ICG) angiography was pointed perpendicularly at the perfusion chamber holding the eye. Images were taken at set time points of 30 seconds, 60 seconds, and 90 seconds, as well as 2, 3, 4, and 5 minutes, after tracer introduction. In angiographically determined high- or low-flow regions (minimum of 5-mm length), the corresponding cornea adjacent to the region was marked (Fig. 3). This size was chosen to be small.

The eye was then unclamped and visualized under a surgical microscope (OMS-90; Topcon Healthcare, Oakland, NJ, USA). The entire TM circumference, except for the previously identified 5-mm high-flow or low-flow stretch, was occluded using cyanoacrylate glue (LOCTITE Super Glue; Henkel Corporation, Rocky Hill, CT, USA) that was gently distributed over the tissue using a BD blunt filling needle (18-gauge, #305180; Becton Dickinson, Franklin Lakes, NJ, USA) (Figs. 1B–1D). Cellulose eye spears (Ultracell, BVI 40415; Beaver-Visitec International, Waltham, MA, USA) were used to touch the TM to remove gross DPBS liquid on the tissue before glue application and then after glue application to confirm that the glue had hardened. If a low-flow region was left open, that eye was designated as a “residual low-flow” experiment. If a high-flow region was left open, that eye was designated as a “residual high-flow” experiment. The eye was then re-clamped onto the perfusion chamber, and the system was flushed again using DPBS. Perfusion was initially continued at 4.5 µL/min.

Given that most of the TM was occluded, IOP always rose. The perfusion system was designed to prevent pressure recordings over 50 mmHg due to sensor design and to avoid damage to the pressure transducers. Therefore, the perfusion rate was adjusted lower to maintain an IOP under 50 mmHg if needed (Fig. 2, where a representative tracing with arrows indicate flow-rate reductions to maintain the eye under 50 mmHg). When a new baseline was reached, the system was flushed with ICG (Cardiogreen, 0.4%; Sigma-Aldrich, St. Louis, MO, USA) with the anterior chamber open to atmospheric pressure. ICG AHO angiography was performed in the same manner as described above for fluorescein. After the experiment, the eyes were unclamped again and inspected under the microscope to ensure that the cyanoacrylate glue TM occlusion stayed intact.

### Histology

After the perfusion study, some of the anterior segments were placed in 4% paraformaldehyde (PFA) in PBS (Thermo Fisher Scientific) overnight at 4°C. The anterior segments were then cryoprotected in 30% sucrose (Thermo Fisher Scientific) dissolved in PBS for 1 day. Wedges, including the TM, were grossly cut under a surgical microscope from eyes that included baseline high-flow TM, baseline low-flow TM, residual high-flow TM after glue, and residual low-flow TM after glue. These tissue wedges were mounted in O.C.T. Compound (Thermo Fisher Scientific). Cryosections 10 µm thick (Leica Biosystems, Vista, CA, USA) were cut, mounted onto Superfrost Plus slides (VWR International, Radnor, PA, USA), dried, taken through ethanol steps (70%–100%) followed by xylenes, stained with hematoxylin and eosin (Epredia, Kalamazoo, MI, USA), and mounted with a coverslip (Sigma-Aldrich). Tissue-section images were captured using a digital slide scanner (NanoZoomer S360 Digital Slide Scanner, 0.5-µm/pixel resolution; Hamamatsu Phototonics, Bridgewater, NJ, USA).

### Data Collection and Statistical Analysis

**Figure 4.**
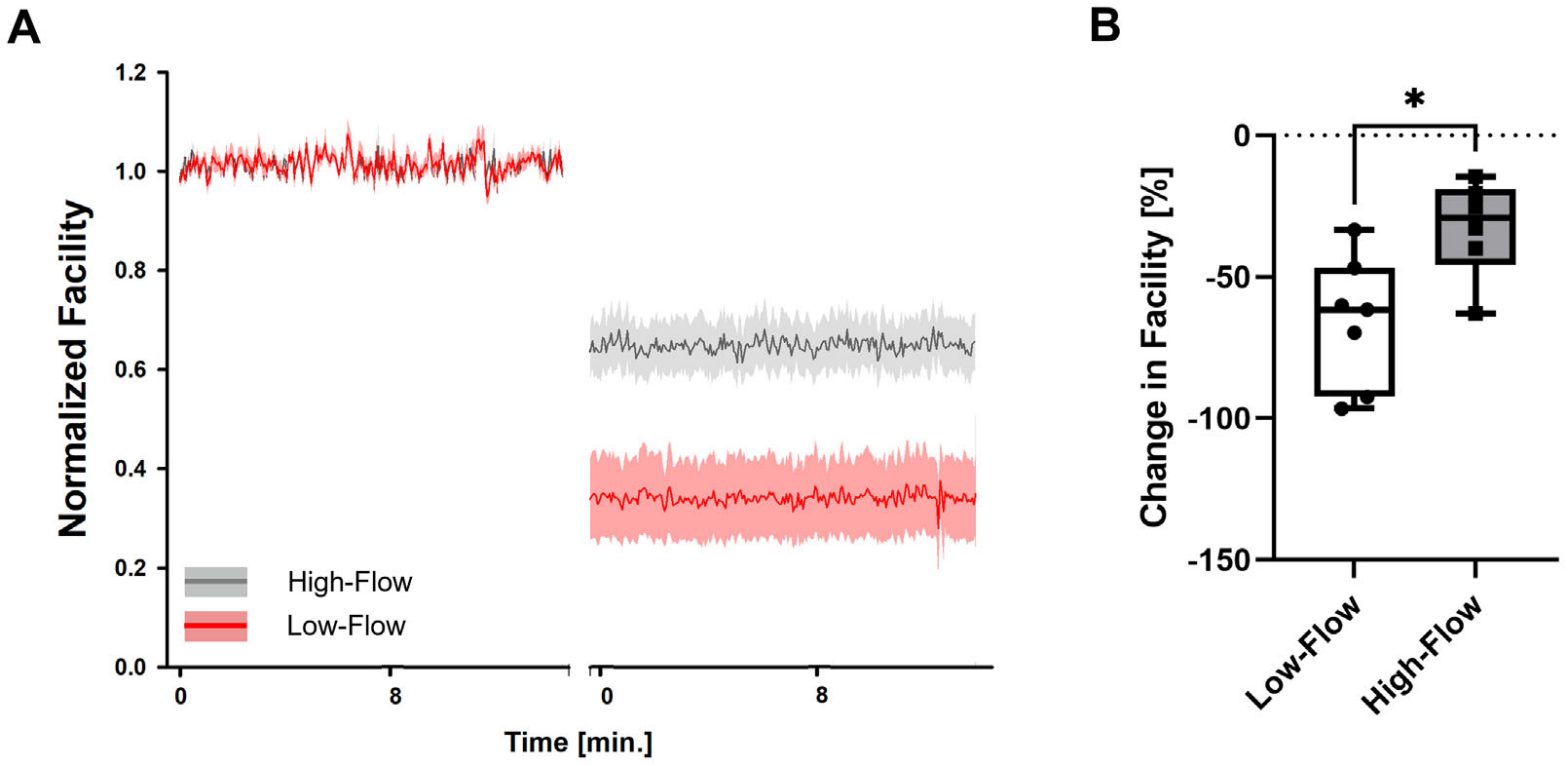
**(A)** Data segments of normalized outflow facility during baseline and after partial TM occlusion being used for the analysis. Both groups exhibited stable conditions. The high-flow group is shown in *gray*, the low-flow group in *red*. Data are shown as mean ± SD (*shaded area*). The time gap between the analyzed stretches has been edited to enhance the readability of the figure. (**B**) Change in facility after partial TM occlusion. Facility decreased by 32.66% ± 19.53% in the high-flow group and by 66.57% ± 23.03% in the low-flow group (*P* = 0.032). The box-and-whiskers plot shows the median and 25th and 75th percentiles (*box*), the data point minimum/maximum (*whiskers*), and all remaining data points (*dots*).

All data were recorded electronically using iPerfusion software. Data were downsampled to ∼1 Hz and subsequently analyzed in LabChart 8.0 (ADInstruments, Sydney, NSW, Australia). For each eye, 15-minute stretches of the stable baseline conditions were used for statistical analysis. Each eye was normalized to its own baseline, and those normalized values were averaged across all study eyes.^19^ Figure 2 shows an example of the analyzed data segments during the course of the experiment. Figure 4 shows the average (and standard deviation) of all normalized segments of all experiments. For each eye, the data were normalized to a short 30-second stretch at the beginning of the baseline period. Comparisons between groups were performed using two-sided *t*-tests (Systat SigmaPlot 12.0; Inpixon, Palo Alto, CA, USA). Changes within one group were assessed using paired *t*-tests. All data are presented as mean ± SD if not stated otherwise. *P* < 0.05 was considered significant.

## Results

Fourteen porcine eyes were used for this study, seven eyes each for the residual low-flow and residual high-flow groups. Fluorescein aqueous angiography was used to determine baseline low- and high-flow regions and to identify the parts of the TM not to be occluded using cyanoacrylate glue (Fig. 3, Supplementary Fig. S1). Baseline outflow facilities were 0.320 ± 0.08 and 0.328 ± 0.10 µL/min/mmHg (*P* = 0.676), respectively, in eventual residual high-flow and residual low-flow eyes before TM occlusion, demonstrating no statistically significant difference. After partial TM occlusion, facility in the residual high-flow group decreased from 0.320 ± 0.08 µL/mmHg/min to 0.209 ± 0.07 µL/mmHg/min (*P* < 0.001), and facility in the residual low-flow group decreased from 0.328 ± 0.10 µL/mmHg/min to 0.114 ± 0.08 µL/mmHg/min (*P* < 0.001) (Table). This demonstrates that the occlusion was effective in lowering AHO facility in both groups.

**Table.** Aqueous Humor Outflow Quantitative Measurements Before and After TM Occlusion

	Group		
	Residual Low-Flow	Residual High-Flow	*P* (High- vs. Low-Flow)
*n*	7	7	—
Baseline IOP (mmHg)	15.32 ± 5.55	14.97 ± 3.30	0.888
Baseline facility (µL/mmHg/min)	0.328 ± 0.10	0.320 ± 0.08	0.676
IOP after occlusion (mmHg)	31.03 ± 8.51	21.30 ± 6.57	0.034
Facility after occlusion (µL/mmHg/min)	0.114 ± 0.08	0.209 ± 0.07	0.035
Facility change (%)	–66.57 ± 23.08	–32.66 ± 19.53	0.032
Perfusion rate after occlusion (µL/min)	3.03 ± 1.96	4.36 ± 0.89	0.127

To compare the residual low-flow and residual high-flow groups, aqueous humor outflow parameters were then evaluated (Table). Although the perfusion flow rate had to be reduced in some eyes (two in the residual high-flow group and three in the residual low-flow group) after the TM occlusion, the post-occlusion perfusion flow rates between the residual high-flow and residual low-flow groups were not statistically significantly different (3.03 ± 1.96 µL/min for the residual low-flow group vs. 4.36 ± 0.89 µL/min for the residual high-flow group; *P* = 0.127). Despite the absence of a statistically significant difference, IOP should be compared cautiously between groups. After occlusion, residual low-flow eyes had a higher IOP than residual high-flow eyes (31.03 ± 8.51 mmHg vs. 21.30 ± 6.57 mmHg; *P* = 0.034).

A more precise way to compare the two groups without having to consider post-occlusion flow rates is to compare the outflow facility (calculated as perfusion rate divided by IOP) between the two groups. Here, the residual high-flow group showed a greater outflow facility after partial TM occlusion compared to the residual low-flow group (0.114 ± 0.08 µL/mmHg/min for the residual low-flow group vs. 0.209 ± 0.07 µL/mmHg/min for the residual high-flow group; *P* = 0.035). Evaluated as normalized outflow facility, stable baselines were seen in both residual low-flow and residual high-flow eyes (Fig. 4A). The residual low-flow group showed a significantly greater percent decline in outflow facility after occlusion compared to the residual high-flow group (66.57% ± 23.08% vs. 32.66% ± 19.53%; *P* = 0.032) (Fig. 4B).

**Figure 5.**
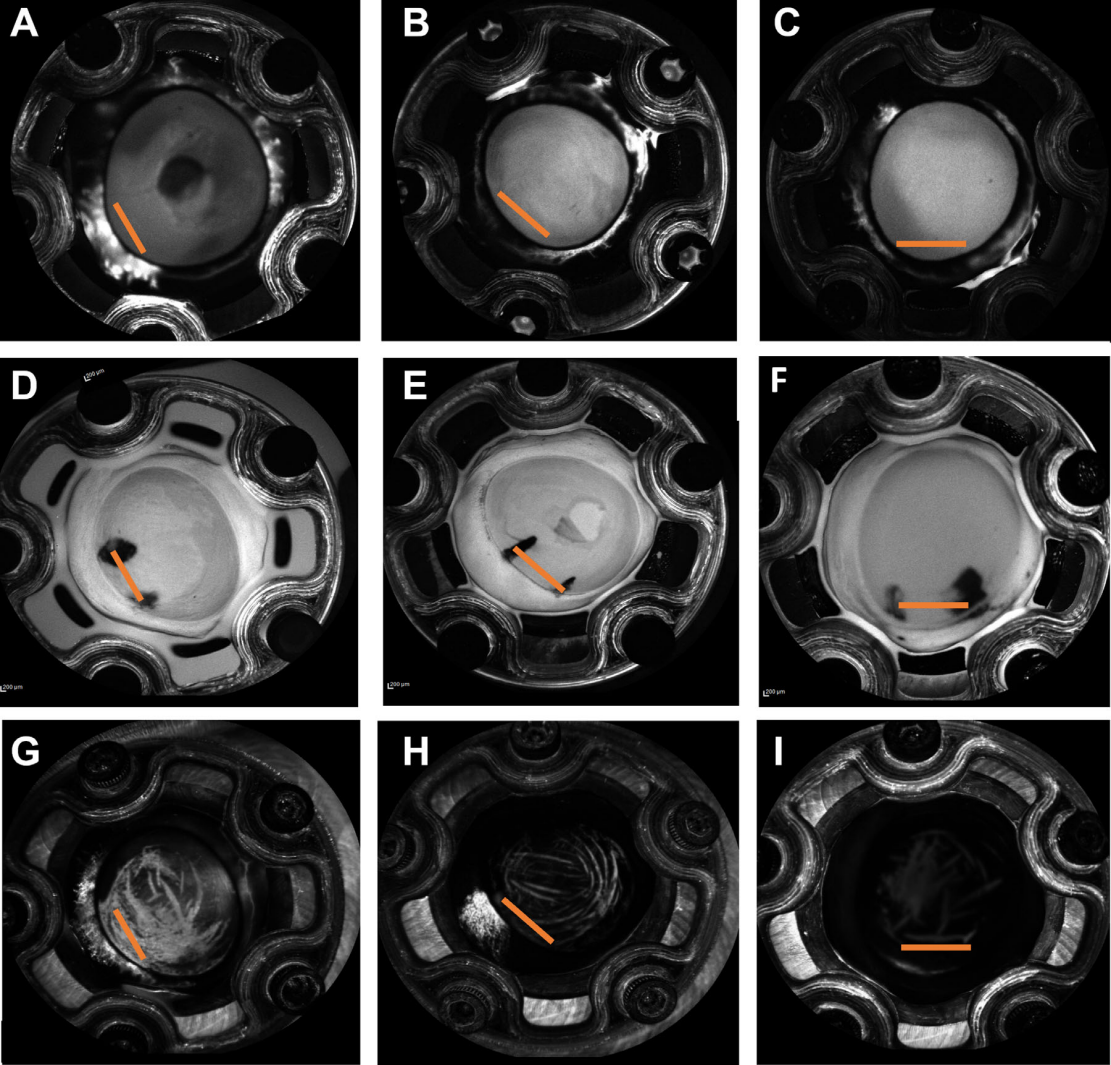
Different ICG AHO angiography patterns after partial TM occlusion (representative example from three eyes). Panels **A**, **D**, and **G**; panels **B**, **E**, and **H**; and panels **C**, **F**, and **I** were acquired from the same eye and are oriented in the same way. Panels **A** to **C** show fluorescein angiography patterns used to discern high- and low-flow regions (all images taken at 90 seconds). Panels **D** to **F** show the open TM stretches as determined by prior fluorescein angiography (region between *black pen line marks*). Three representative and different AHO patterns were observed using ICG angiography: (**G**) ICG signal extending beyond the open TM; (**H**) localized AHO in the region of the open TM, even though a slight offset was observed; and (**I**) almost no AHO signal, which was observed in only one eye and led to very low facility values. The *orange lines* facilitate comparison among the eyes.

**Figure 6.**
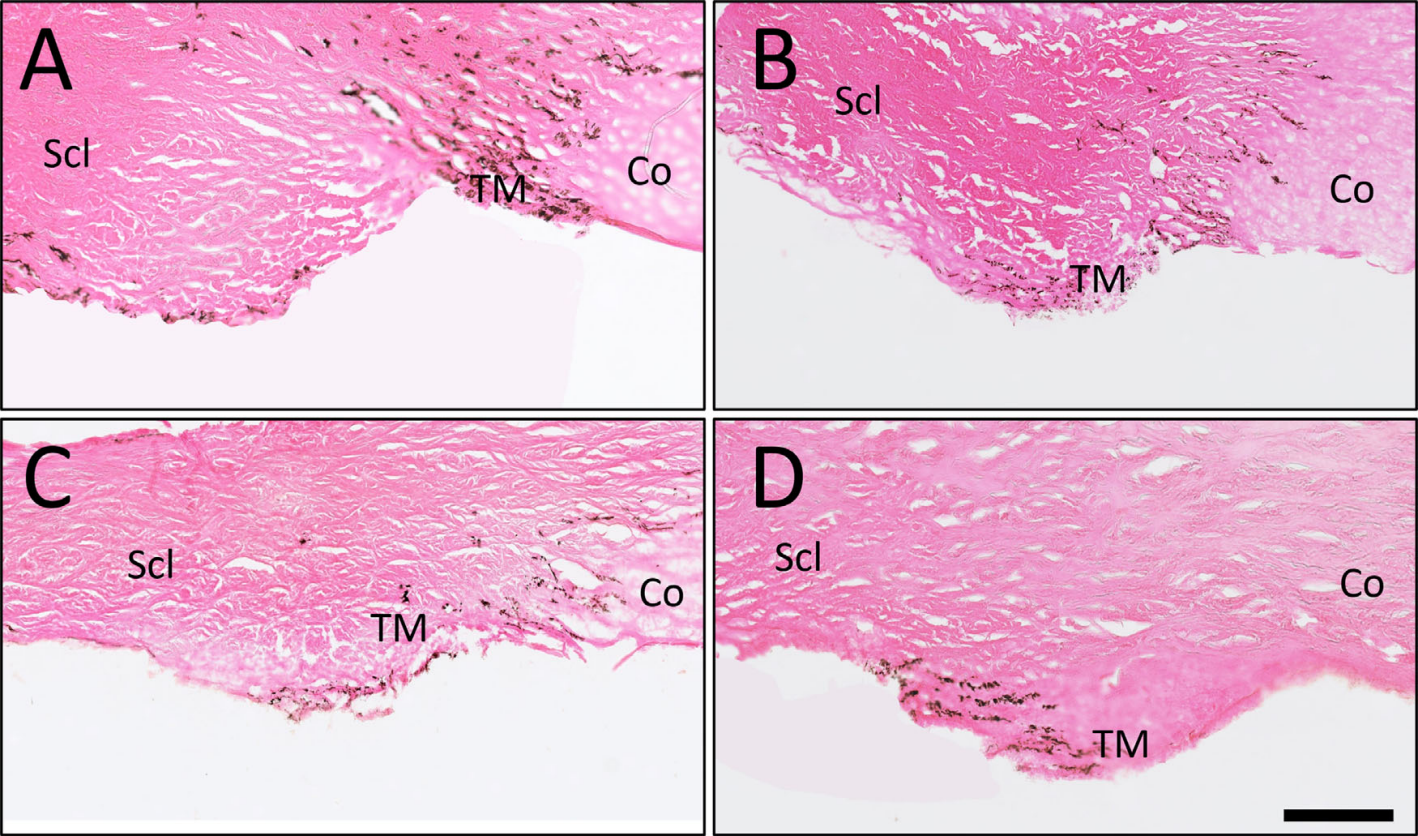
Trabecular meshwork histology from (**A**) a baseline high-flow TM region, (**B**) a baseline low-flow TM region, (**C**) a residual high-flow TM region after glue, and (**D**) a residual low-flow TM region after glue. *Scale* *bar*: 500 µm. Co, cornea; Scl, sclera.

After occlusion, ICG angiography was also performed, and three different patterns were seen. All except for one, eyes demonstrated continued flow near the residual open TM regardless of baseline outflow pattern (Fig. 5). In the first pattern seen, the ICG angiography signal was centered within the residual TM opening and extended perilimbally (Figs. 5A, 5D, 5G). In the second pattern, the residual ICG angiography signal was near the residual TM opening but off to one side (Figs. 5B, 5E, 5H). One residual low-flow eye showed almost no ICG outflow after the occlusion (Figs. 5C, 5F, 5I). Histologically, TM tissue in all conditions (baseline high-flow, baseline low-flow, residual high-flow after gluing, and residual low-flow after gluing) appeared grossly similar and normal (Fig. 6).

## Discussion

In the present study, we evaluated whether different TM segments of an eye demonstrate different local aqueous humor outflow facilities. We chose the TM regions based on aqueous angiographically determined baseline high- and low-flow areas under the idea that localized TM facility differences explain segmental outflow seen using aqueous angiography. Our data support the hypothesis that angiographically determined high- and low-flow regions have different local outflow facilities.

The main finding of the present study is that high- and low-flow regions exhibited different facilities after partial TM occlusion using cyanoacrylate glue. To the best of our knowledge, this is the first attempt to acquire local (segmental) facility measurements for this purpose. However, it should be noted that our approach provides the local outflow facility estimate by occluding the majority of the TM to infer the facility in residual regions. This is as opposed to direct measurement of outflow facility in the regions of interest in an otherwise normal or in vivo eye. A previous study by Hong et al.^26^ investigated outflow facility in a similar setup, using cyanoacrylate-mediated TM-occlusion as a model to study angle closure in porcine eyes. The authors reported no significant change in outflow facility for 90°, 180°, and 270° of occluded TM. This might imply that there is no segmental outflow facility in the eye. However, several differences exist in the experimental design and protocols between these two studies. First, segmental outflow facility may only be observed when smaller TM segments are isolated. Ninety degrees of open TM (in the 270° TM occlusion condition) corresponds to approximately twice the size of the residual TM opening in this present study. Also, Hong et al.^26^ showed a numerical decrease in outflow facility (∼10%–20%) for the 270° occlusion group compared to the unoccluded group, although it did not reach statistical significance. This lack of statistical significance may be explained by an insufficient sample size (four eyes per group). A post hoc analysis of the data of Hong et al.^26^ gave a power below 0.5 to detect a 20% decrease in facility. Furthermore, the choice of location for the residual open TM in Hong et al.^26^ was not guided by baseline-determined high- and low-flow segmental regions. Thus, all TM residual openings in that earlier study could have been baseline high flow, low flow, or mixed flow. In our study, we specifically isolated and only compared known high- to low-flow regions.

The result of varying outflow facility in different parts of the TM is important because it has been hypothesized that segmental AHO is due to differences in facility as opposed to an intrinsic TM biological property that is unrelated to outflow facility. Biological differences in protein expression and extracellular matrix have been shown comparing high- and low-flow TM regions by multiple groups using different methods.^3^^,^^5^^–^^7^^,^^9^ Thus, TM cells could theoretically metabolize tracers in a segmental fashion to give the impression of segmental AHO. Alternatively, segmental difference in the extracellular matrix could differentially bind fluorescent tracers to give the appearance of segmental AHO again. The results of this paper indicate that such alternative explanations of segmental AHO in tracer studies are less likely and that differences in outflow facility across the TM explain segmental outflow.

Local differences in TM facility also have potential clinical implications. TM-targeted MIGS decrease IOP by bypassing the TM and lowering resistance through placement of a high-facility device in a focal TM area. The current clinical practice treats the TM as a uniform tissue; that is, all segments are assumed to have the same facility. Thus, local outflow facility is not considered, and surgery can be performed anywhere. However, if the TM resistance is not distributed in a uniform manner, performing trabecular bypass MIGS in different locations may yield different IOP-lowering effects. More specifically, it could be desirable to specifically target areas of the eye with the lowest facility to maximize the overall effectiveness of the procedure. Although the goal of targeted MIGS has been proposed^25^ and results herein provide preclinical data from postmortem eyes to that effect, definitive clinical proof is still needed.

Overall, it is also clear that AHO is no longer a static and simple concept that can be reduced to one number in the Goldmann equation. In addition to the finding of varying outflow facility in different parts of the TM, another example of this is the observation that the conventional outflow pathways and the TM can demonstrate physiological behaviors that can shift and change over time. For example, in vitro studies have shown that segmental outflow can change from low to medium to high flow over time.^31^ Also, studies in human patients have shown dynamic shifting of AHO from one part of the eye to another for yet unclear reasons.^32^ Observations from human patients could arise from biological regulation at the TM or distal to the TM.

Another intriguing observation in the present study is the different ICG angiography patterns observed after partial TM occlusion (Fig. 5). As expected, ICG flow was mostly located around the residual open TM and was seen in nearly all cases after TM occlusion. However, there was variability in that appearance. Some eyes displayed a slightly offset ICG signal relative to the open TM stretch that did not spread laterally (Figs. 5E, 5H). Other eyes showed ICG signal nicely centered around the middle of the residual TM opening with extension beyond (Figs. 5D, 5G). Tracer spread beyond the extent of a TM opening has been seen by other researchers.^12^^,^^13^ It is important to note that porcine eyes do not possess a continuous, circular Schlemm's canal equivalent to that of humans^33^ but instead a discontinuous and more tortuous structure referred to as the angular aqueous plexus, or Schlemm's canal-like pathways. This irregular distal anatomy potentially explains the variable ICG angiographic patterns that were seen from residual open TM regions after occlusion elsewhere. Further studies are needed to investigate for possible post-trabecular or distal differences. For the study of TM outflow, porcine eyes are considered a valid model nevertheless, as their physiology and, more importantly, facility are comparable to those of human eyes.^33^^–^^35^

A number of limitations must be discussed for this study. First, cyanoacrylates, although routinely used for medical care, can differ in the heat they generate during polymerization.^36^ In this study, this may have impacted the tissue; however, the gross structure of the non-glued outflow pathways appeared intact. Further, both experimental groups in this study (residual high-flow and residual low-flow) experienced the glue equally such that heat-induced changes to tissue had a control. Another important limitation of the present study is the lack of pressure in the distal outflow system (i.e., episcleral veins, scleral veins, and collector channels) and also the lack of neuronal tone in the distal outflow system. The distal outflow system is segmental by nature, and recent evidence suggests that it may be regulated, at least in part, by neuronal mechanisms.^37^^–^^40^ The role of these neuronal mechanisms in regulating conventional outflow in vivo remains to be elucidated.

In summary, the present study further supports the long-standing assumption that high- and low-flow segments of the trabecular meshwork exhibit different outflow facilities. Future studies (including studying human eyes in the laboratory and in vivo) are needed to understand the biological cause for different outflow facilities through the conventional outflow pathways and to determine whether these findings can be translated to improve surgical procedures of the TM.

## Supplementary Material

Supplement 1
