# Supplementary material for: Differences in Outflow Facility Between Angiographically Identified High- Versus Low-Flow Regions of the Conventional Outflow Pathways in Porcine Eyes
Source: Invest Ophthalmol Vis Sci. 2023 Mar 20;64(3):29. doi: 10.1167/iovs.64.3.29 (PMC10043501; doi:10.1167/iovs.64.3.29)
Supplement: Supplement 1 [file iovs-64-3-29_s001.pdf]

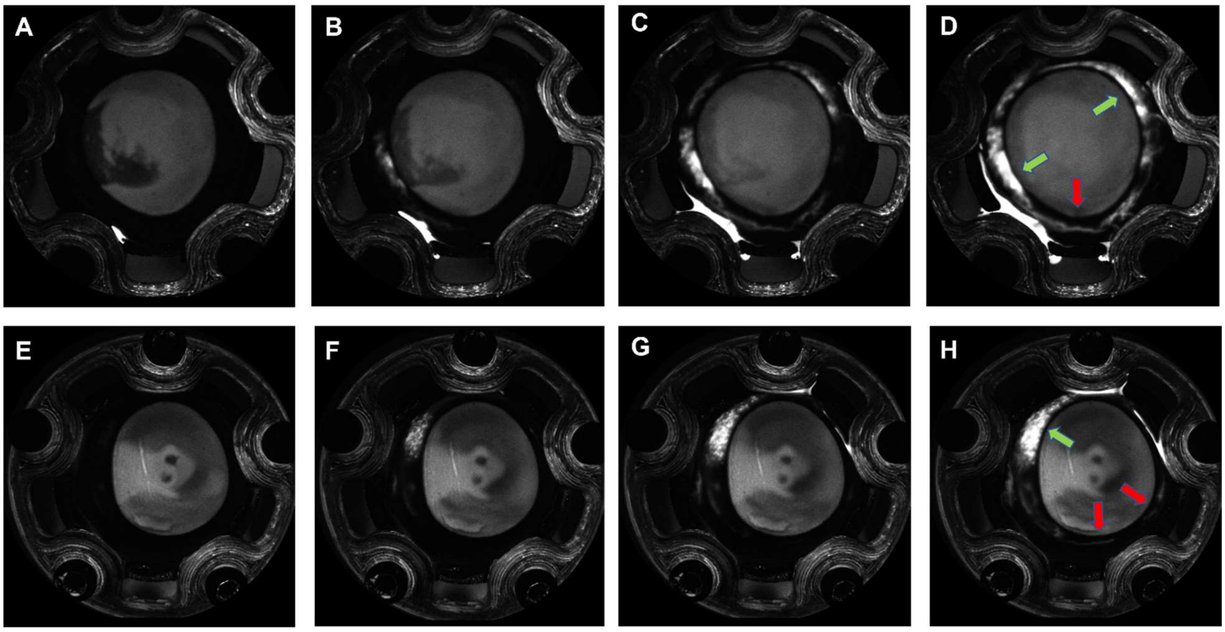

Supplemental Figure 1. Additional Fluorescein angiography images in two different eyes. Images immediately taken after (A/E), at 30 seconds (B/F), 60 seconds (C/G) and at 90 seconds (D/H) post-tracer introduction. Post-limbal high- and low flow regions could be identified unambiguously in both eyes and are marked using green (high-flow) and red (low-flow) arrows in the images taken at 90 seconds (D/H).

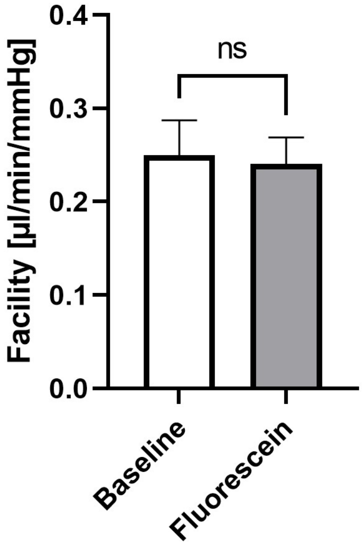

Supplemental Figure 2. Outflow facility before and after fluorescein angiography has been performed during preliminary experiments (n=3).
